# Amalgam Experiments

**Published:** 1871-12

**Authors:** S. P. Cutler


### SELECTED ARTICLES.

---

### ARTICLE VI.

### *Amalgam Experiments.*

BY S. P. CUTLER, M. D., D. D. S.

I took of good amalgam twenty-four grains and mixed with mercury, usual way, and expressed through buckskin to the proper dryness, then washed in alcohol and weighed. Thirteen grains of mercury had been retained. After two weeks, I weighed again and found a loss of two grains, the whole weighing thirty-five grains, a little less than half as much mercury as of silver and tin. The small amount of evaporation of mercury in thirty-five grains of amalgam is so insignificant, that no possible harm could take place by the use of even that amount at one sitting, which would be sufficient to fill several quite large cavities, though in some extreme cases double the amount or more may be used in one tooth, even then no danger of salivation need be apprehended in any subject.

By applying heat with the blow pipe the dry lump became soft and plastic, as when first mixed, only a little more brittle.

The chemical affinity betweed the mercury, tin, and silver, is very strong and not easily broken up, and is known as the law of adhesive affinity or that of wetting, which is explained in this way: Any liquid whose affinity or cohesion is greater for any solid than the cohesive attractions of its own molecules, a perfect solution of saturation takes place.

Again, any liquid whose affinity or cohesion for any solid is more than half what it is among its own molecules, simply wetting takes place, nothing more.

Any liquid whose affinity for a solid is less than one-half what it is among its own molecules no wetting takes place at all, as mercury and glass, porcelain, and many other solids, as mercury and bone or dentine demand. The reason why water and oil or mercury do not unite depends on this same law. The word adhesion and cohesion may both be used in the above explanation. This specimen softened by heat, and gave up the mercury, though rather explosively, so that I could not ascertain the final result by the blow pipe. In fact, this is a feeble fulminate.

Pure silver amalgam.

Twenty-four grains of pure silver filings combined with 28 grs. of mercury when expressed through buckskin as in ordinary use of amalgam in filling teeth. It will be seen that pure silver requires more than double the amount of mercury to amalgamate it than ordinary tin and silver combined.

Pure silver does not make a tough and tenacious mass, only slightly adherent. Washing with alcohol does not produce any dark color as in ordinary amalgam.

No loss or subsequent hardening takes place by standing for six days, weather hot. On applying heat with the blow pipe, light colored fumes began to pass off and continued to escape frequently in sudden gusts, and continued up to a red heat, at which temperature I kept it for a few minutes until all fumes ceased. It did not become soft and plastic at all, like ordinary amalgam, but remained hard during the process, becoming light yellow, and did not lose any of its cohesive properties by heating. There was a loss of twenty-four grains out of the twenty-eight of mercury.

Amalgam of pure tin filings.

Twenty-four grains of pure tin filings took up fourteen grains of mercury, forming a mass in all respects similar to ordinary amalgam: did not lose in weight by standing several days. Washing with alcohol produced about the same amount of dark matter as common amalgam. There was no subsequent hardening from the first melting on charcoal, for fifteen minutes caused no loss in weight, no fumes escaped. After cooling, the mass was found to be quite hard and tough, polished well, and pure white.

The probability is that amalgam would not harden sufficiently in the tooth to make a filling.

Silver coin amalgam, or almalgam of olden time.

Twenty-four grains of coin fillings, takes up thirty-two grains of mercury to amalgamate. After several days standing I found the mass hard and firm, had lost scarcely a grain in weight. On applying heat it did not become at all plastic, but remained hard. I continued the heat up to redness, for at least twenty minutes before the mercury fumes ceased passing off; on weighing, I found nearly all the mercury had escaped; the lump still remained hard and firm though more brittle than before applying heat.

Now the question is, what holds the filings together, when all but a trace of the mercury has been dispelled by heat? Washing with alcohol produced dark matter.

PURE GOLD AMALGAM.

Twenty-four grains of pure gold filings takes up fifteen grains of mercury to amalgamate. It instantly becomes white like pure silver amalgam; on standing several days there was no loss of weight, neither did it set or harden to any extent, in consequence it will not answer for filling teeth. There was no stain from washing. On applying heat with the blow pipe, up to redness, for five minutes, it became yellow and lost all the mercury and two grains more. The filings were not melted, and the mass much tougher than before heating. On melting the gold there was no more loss of weight, the gold apparently as pure, and worked as well as though there had never been any mercury in combination.

EIGHTEEN CARAT GOLD AMALGAM

Twenty-four grains of eighteen carat gold *silver alloy*

takes up twenty grains of mercury to amalgamate. It does not become so instantaneously white as pure gold, neither was the white so intense.

On washing with alcohol there was scarcely any stain. After several days drying there was no loss by evaporation, the mass becoming quite hard and brittle. This amalgam will answer a very good purpose in some cases for filling teeth. I have had a filling of this kind in one of my back teeth, for five or six years, now pefectly sound, only dark on the surface owing to the silver it contains.

On applying heat to the specimen, and raising up to redness for five minutes, all the weight of mercury disappeared still leaving a hard firm mass, which takes a fine polish and has a golden color.

AMALGAM OF CADMIUM.

Twenty-four grains of cadmium filings amalgamate with forty-two grains of mercury, and sets or hardens almost instantaneously if quite dry, even without sqeezing through buck skin. On rubbing in the palm of the hand the finger becomes black, and on washing with alcohol about the same amount of dark matter is produced as in common amalgam.

On drying for several days it looses nothing in weight

While rubbing up in the palm of the hand a considerable amount of heat is developed, I presume, from the rapid conversion of the liquid mercury into a solid, the latent force of the fluid mercury being transformed into sensible heat.

This amalgam is almost certain to destroy the nerves in all teeth filled with it, turning the tooth yellow and staining allthe balance at the line of the gum the same color. This yellow stain is the result of a yellow soluble oxide, the fillings remaining white owing to the solubility of the oxide.

The specimen lost one grain after several days drying. On melting on charcoal it does not evaporize or loose in weight, and melts at a very low heat, and remains fluid a long time and retains the white color.

After being melted it is very tough and may be hammered or rolled into a plate, and cuts easily with a knife. This amalgam may be found valuable for some purposes out of the mouth.

There are some dentists still using this amalgam to my knowledge for filling teeth. It should never be used in the mouth under any circumstances.

By heating cadmium sufficiently hot, yellow vapors of the oxide of the metal are formed. Mercury unites readily and hardens with this metal, but does not harden to any extent when united with any of the other simple metals experimented with. Why this is so, I am unable to say, unless it is from the fact that mercury and cadmium are the only two metals whose combining volumns of vapor are double that of any other elementary bodies. All good amalgams must be composed of at least two metals besides mercury. None of the above metals decompose water to any extent under ordinary circumstances.

I experimented with platinum and mercury, also aluminum and mercury, but found that neither of them formed metalic amalgams with mercury, only oxides of which I shall have occasion to speak sometime, under another head, more especially that of aluminum. This metal is one of the most selfish and unfriendly of all known metals, refusing to unite or form any metallic alliance with any other metal. This is a great and wise provision in nature's economy, this metal forming as it does the basis of most soils; the earth would be but a barren waste if this metal held strong affinities for other metals; when the earth was in a melted state alloys would have formed without end so as to have prevented its capacity for uniting with oxygen to form clays.

The metals suitable for amalgams are reduced down to three, silver, gold, and tin, mercury forming the amalgamating metal. Tin and silver make the best, silver and gold answer very well, gold and tin is too brittle and hard for practical purposes it does not harden at all when amalgamated; none of the other metals will answer as they would not stand in the mouth.—*Canada Journal of Dental Science.*

